# Comparative Analysis of *Felixounavirus* Genomes Including Two New Members of the Genus That Infect *Salmonella* Infantis

**DOI:** 10.3390/antibiotics10070806

**Published:** 2021-07-02

**Authors:** Rocío Barron-Montenegro, Rodrigo García, Fernando Dueñas, Dácil Rivera, Andrés Opazo-Capurro, Stephen Erickson, Andrea I Moreno-Switt

**Affiliations:** 1 Laboratorio de Investigación en Agentes Antimicrobianos, Departamento de Microbiología, Facultad de Ciencias Biológicas, Universidad de Concepción, Concepción 4030000, Chile; rbarron@udec.cl (R.B.-M.); andopazo@udec.cl (A.O.-C.); 2 Escuela de Medicina Veterinaria, Facultad de Agronomía e Ingeniería Forestal, Facultad de Ciencias Biológicas, Facultad de Medicina, Pontificia Universidad Católica de Chile, Santiago 7550000, Chile; 3 Millennium Initiative for Collaborative Research on Bacterial Resistance (MICROB-R), Lo Barnechea, Santiago 7690000, Chile; dacil.rivera@unab.cl; 4 Laboratorio de Microbiología, Instituto de Biología, Pontificia Universidad Católica de Valparaíso, Valparaíso 2340000, Chile; rodrigo.garcia@emory.edu; 5 Department of Biology, Emory University, Atlanta, GA 30322, USA; 6 Escuela de Medicina Veterinaria, Facultad de Ciencias de la Vida, Universidad Andres Bello, Santiago 8320000, Chile; ferdu26@gmail.com; 7 Laboratory Corporation of America Holdings, New Brighton, MN 55112, USA; erickss@labcorp.com

**Keywords:** bacteriophages, genomes, *Felixounavirus*, *Salmonella* spp., *Salmonella* phages, comparative genomics

## Abstract

*Salmonella* spp. is one of the most common foodborne pathogens worldwide; therefore, its control is highly relevant for the food industry. Phages of the *Felixounavirus* genus have the characteristic that one phage can infect a large number of different *Salmonella* serovars and, thus, are proposed as an alternative to antimicrobials in food production. Here, we describe two new members of the *Felixounavirus* genus named vB_Si_35FD and vB_Si_DR94, which can infect *Salmonella* Infantis. These new members were isolated and sequenced, and a subsequent comparative genomic analysis was conducted including 23 publicly available genomes of *Felixounaviruses* that infect *Salmonella*. The genomes of vB_Si_35FD and vB_Si_DR94 are 85,818 and 85,730 bp large and contain 129 and 125 coding sequences, respectively. The genomes did not show genes associated with virulence or antimicrobial resistance, which could be useful for candidates to use as biocontrol agents. Comparative genomics revealed that closely related *Felixounavirus* are found in distinct geographical locations and that this genus has a conserved genomic structure despite its worldwide distribution. Our study revealed a highly conserved structure of the phage genomes, and the two newly described phages could represent promising biocontrol candidates against *Salmonella* spp. from a genomic viewpoint.

## 1. Introduction

Salmonellosis is the most common foodborne and zoonotic disease reported worldwide [1]. Estimation of salmonellosis cases includes 93.8 million cases of diarrhea and 155,000 deaths annually [2]. In addition, the economic impact of foodborne illness is estimated at $90.2 billion in the United States, and the cost associated with *Salmonella* spp. in foodborne illnesses is calculated to be around $5.4 billion [3]. There are two species described for this genus, *Salmonella bongori* and *Salmonella enterica,* which are further classified by the White-Kauffmann-Le Minor scheme into more than 2600 serovars [4]. Currently, *S.* Typhimurium, *S.* Enteritidis, and *S.* Infantis are the most prevalent *Salmonella* serovars worldwide [5]. Human salmonellosis cases and outbreaks are attributed to contact with infected animals such as livestock and, predominantly, by the consumption of contaminated food [6]. To reduce food contamination with *Salmonella*, several interventions are conducted during food production; despite this, incidents such as recalls and outbreaks are common, therefore, novel strategies to control *Salmonella* are necessary, for instance, by utilizing bacteriophages (or phages) [7]. Phages are viruses that infect specific bacteria and are positioned as a biotechnological tool capable of rapidly controlling bacterial growth, and even controlling bacteria resistant to antimicrobials. This last point is of great relevance for controlling new serovars and resistant bacteria emerging from animal production systems.

Bacteriophages are the most abundant entities on earth, with an estimated 10^31^ viral particles in the ocean [8]. Phages were first described by Frederick Twort in 1915, who described glassy and transparent colonies in micrococci cultures, called ‘ultra-microscopic virus’ [9]. Later in 1917, Felix D’Herelle described these viruses as an “invisible microbe” with antagonistic activity against *Shigella* spp. and used the term ‘bacteriophage’ [10]. Viral taxonomy is regulated by The International Committee of Taxonomy of Viruses (ICTV), which classifies phages according to their morphology, physicochemical properties, nucleic acid content (DNA or RNA), and genomic information [11]. The *Myoviridae* family has dsDNA genomes and a morphology characterized by an icosahedral capsid that connects a neck, where the short tail fibers and the tail emerge, while the latter is composed of the tape-measure protein that surrounds the tail tube, and finally is covered by the external contractile sheath, ending at the base plate, from where the long tail fiber (LTF) emerges [12]. Bacteriophage Felix O1 was one of the first *Myoviridae* reported in 1943 by Felix D`Herelle and Callow [13]. In 2011, it was classified as the type species of the *Felixounavirus* genus proposed by the ICTV [14]. Importantly, phages of *Felixounavirus* genus have been shown to infect several different types of *Salmonella* serovars [15,16]. Further reports of phages of the *Felixounavirus* genus have shown their potential for infecting *Salmonella* of numerous serovars, including descriptions of isolations from several countries such as Russia, the United States, and Chile, which were recovered in samples from different sources: chicken and bovine feces, sewage, and farm soil [16,17,18,19]. Moreover, these reports have shown that *Felixounaviruses* are strictly lytic, displaying promising potential to be used for the biocontrol of *Salmonella* spp. [20]. For instance, under different growth conditions, a *Felixounavirus* isolate named UAB_Phi87 obtained from farms in Spain caused a significant reduction of *Salmonella* Enteritidis of about 5.4 logs CFU/mL and a reduction of 5.3 logs CFU/mL of *Salmonella* Typhimurium in liquid cultures [21]. This phage was also used in a cocktail with two other phages and tested on different food matrixes, such as pig skin, in which it reached a reduction of *S.* Enteritidis and *S.* Typhimurium of 2 and > 4 logs/cm^2^, respectively [22]. While the reduction of logs (CFU/mL) is widely reported in the literature when reduction effectiveness is compared with other phages or other antimicrobials, the interactions between phage and bacteria are different in comparison with antimicrobials; interaction is mainly physical between the phage and their host, and other factors are involved, such as the concentration of bacteria and phage or the multiplicity of infection (MOI) [23]. A different study reported that *Felixounavirus*, when microencapsulated and applied via dry spraying for oral application, an alternative application, was also remarkably stable [24]. There are products for control of pathogens that use phages of this genus, such as IntestiPhage for control of gastrointestinal disease developed at Eliava Institute in Georgia which used Mushroom phage, SalmoFRESH, which includes phage BSP101 and five other phages and is used for biocontrol of *Salmonella* in vegetables [25,26]. Overall, *Felixounavirus* have been reported in several countries and they are well adapted to *Salmonella* with several reports of their lytic potential, which represents an interesting alternative intervention to reduce the load of this important foodborne pathogen. Hence, this study aimed to characterize the genomes of two new members of the *Felixounavirus* genus that infect *Salmonella*, vB_Si_35FD and vB_Si_DR094, and to conduct a comparative genomic analysis with previously described *Felixounavirus* to understand the worldwide diversity and relatedness of *Felixounavirus* with tropism for *Salmonella*. We hope that the background provided by this study will contribute to the robustness of the genetic information on phages of the genus *Felixounavirus*, which could have significant relevance for the biocontrol of *Salmonella*.

## 2. Results and Discussion 

### 2.1. Phages vB_Si_35FD and vB_Si_DR094 Represent New Members of the Felixounavirus Genus that Infect a Large Number of Salmonella serovars

**Figure 1:**
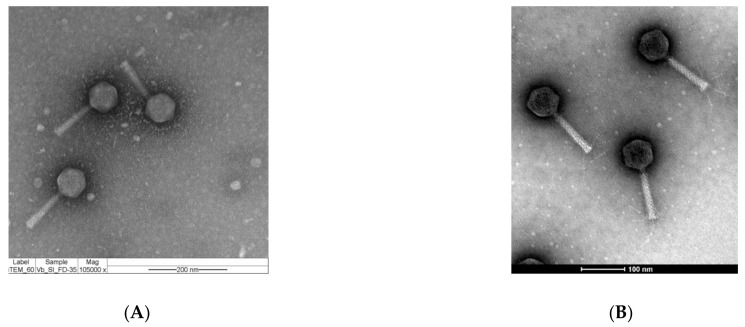

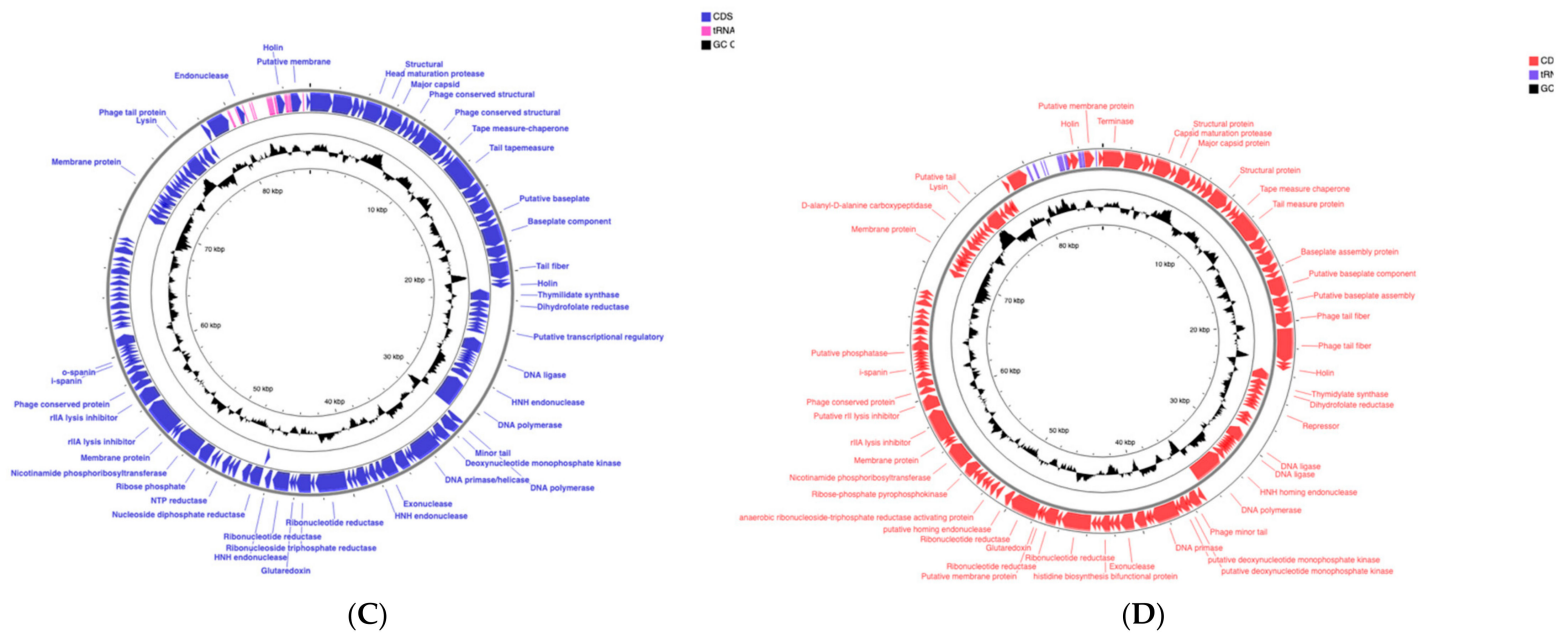
Morphological and genomic characterization of sequenced phages. (**A**) Image obtained by TEM, vB_Si_35FD, (**B**) vB_Si_DR094, both phages exhibit icosahedral capsids and long straight tails, which is consistent with the morphology of the *Myoviridae* family. (**C**) Visualization of the complete genome of vB_Si_35FD sized 85 kb with 129 identified CDS (blue); most of the genes were transcribed from the forward strand (external CDS) and 19 tRNAs (pink); in black is the G+C content that, on average, was 38.9%. (**D**) vB_Si_DR094 genome visualization, sized 85.7 kb with 125 identified CDS (red); in purple are marked 20 tRNAs, and the G+C% on average was 39.2% (black).

Transmission electron microscopy (TEM) and genomic characteristics were investigated to classify these two newly sequenced phages. TEM images showed that phage vB_Si_35FD and phage vB_Si_DR094 exhibited an icosahedral capsid with long, straight tails, which is concordant with *Myoviridae* family morphology (Figure 1A,B). The host range analyses showed that Phage vB_Si_35FD has a broader host range in comparison to vB_Si_DR94. Phage vB_Si_35FD is capable of infecting 17 different serovars of *Salmonella enterica* including: Infantis, Virchow, Panama, Heidelberg, Newport, Corvalis, Dublin, Stanley, Agona, Montevideo, Typhimurium, Javiana, Mbandaka, Oranienburg, Choleraesuis, Braenderup, and 4,5,12:i:-. On the other hand, phage vB_Si_DR94 infects 12 different serovars of *Salmonella enterica* including serovars Virchow, Saintpaul, Panama, Montevideo, Infantis, Javiana, Newport, Stanley, Mbandaka, Heidelberg, Choleraesuis, and Oranienburg. Other reports show that phages of *Felixounavirus* genera had the capacity to infect several different serovars of *Salmonella*, as phage vB_Si_SF20-2 can infect 14 different serovars of *Salmonella*, and phage vB_Si_QUI-1 can infect six different serovars [16]. This characteristic is an advantage in their potential use as biocontrol.

Further genome sequencing showed that phage vB_Si_35FD and vB_Si_DR094 contain genomes sized 85,818 and 85,730 bp, respectively (Table 1). The genome sizes found here are in the range of those reported for the *Myoviridae* family, for which a wide range of sizes is described from approximately 50,000 to 150,000 bp [27]. The average G+C content of the genomes was 38.9%, which is lower than the G+C content of their *Salmonella* host (~ 50% of G+C), but similar to previously reported G+C content for *Salmonella* phages [28]. The number of tRNAs in phage vB_Si_35FD was 19 and, in phage vB_Si_DR094 was 20 (Table 1). The number of tRNA-encoding sequences is related to the length of the genome [29]; as for this case, the amount of tRNA may also be related to the codon usage of these phages [30]. The annotation of the phage genomes showed 129 and 125 coding regions for vB_Si_35FD and vB_Si_DR094, respectively. The number of proteins associated with an annotated function initially was 26 and 25, respectively, for both phages. To improve this estimation, we manually curated the annotations from BlastP mapping and then identified each CDS annotated from RAST. This manual annotation allowed us to identify a total of 46/129 function-associated proteins for phage vB_Si_35FD and 56/125 for phage vB_Si_DR094, which highlights the importance of manual annotation for phage genomes.

Furthermore, we observed an organization based on transcriptional modules in both genomes, from the terminase to genes associated mainly with phage structure, such as the capsid and tail genes (Figure 1C,D). Bacteriophages have shown a characteristic structure in their genomes, known as early, middle, late, and lysis genes. This order is presented by the transcription of their proteins, an organization described for some phages such as lambda, T4 and phage FelixO1 [28,31,32]. In this work, we observed the same transcriptional organization, and the first module contained genes coding for structural proteins, classified as late genes. In phage vB_Si_35FD, seven tail-associated proteins were annotated, including tail fibers and baseplate components, while in phage vB_Si_DR094, eight tail-associated proteins were annotated (Figure 1C,D). The tail fibers are responsible for the recognition of bacterial receptors, thus conferring specificity [33]. In the case of phages of the genus *Felixounavirus* that infect *Salmonella*, lipopolysaccharide (LPS) has been described as the bacterial receptor [34]. Additionally, one recent publication reported that the emergence of single nucleotide polymorphism (SNP) in LPS biosynthesis involved proteins when *Salmonella* is exposed to *Felixounavirus* [19], thus reaffirming that LPS represents the bacterial receptor for *Felixounaviruses*. 

The second cluster of genes was identified as related to nucleotide metabolism and DNA replication, known as early genes because they are involved in the first stages of infection. Phages vB_Si_35FD and vB_Si_DR094 contained proteins such as DNA polymerase, DNA ligase, and DNA primase/helicase, all necessary for DNA replication. In both phages, a gene encoding a gluta-redoxin was annotated, which is associated with the reduction of thioredoxin agents involved in deoxyribonucleotide biosynthesis, as reported in the phage T4 [35]. Dihydrofolate reductase and thymidylate synthase were also identified in both phages, in which the dihydrofolate reductase reduces 7,8-dihydrofolate to tetrahydrofolate and acts as a cofactor in the conversion of dUMP to dTMP by the thymidylate synthase enzyme; they are also involved in the synthesis of DNA and RNA acting as precursors [36]. In phages it is seen that the conversion of dNMP to nucleotides is catalyzed by a single broad-substrate-specific enzyme, deoxynucleotide kinase, that, in the phages vB_Si_35FD and vB_Si_DR094, was annotated as deoxynucleotide monophosphate kinase [28]. Additionally, the presence of genes related to nucleotide metabolism appears to be conserved in *Felixounaviruses* [37].

The third genomic module contained genes annotated as homing endonucleases, which were distributed in both phage genomes, including three genes in vB_Si_35FD and two in vB_Si_DR094. Homing endonucleases are site-specific enzymes that break the double strand of DNA allowing the insertion or mobilization of genes, in which T-even-like phage homing endonucleases have been widely described as components of their genomes [38]. In phage FelixO1, six copies of DNA sequences associated with homing endonucleases have been described [28]. In phage T4, the functions of homing endonucleases include a regulatory role in transcription due to their location in the genome; specifically, they are closely related to the promoters of middle or late genes, which are essential for the life cycle of phage T4 [39].

A fourth genomic segment was identified that contains genes associated with bacterial lysis, classified as lysis genes in the final order of transcriptional organization, known as late or lysis genes. This module includes genes encoding the o-spanin and i-spanin proteins, which are involved in the three-step lysis pathway of Gram-negative bacteria, such as *Salmonella* spp. [40]. The i-spanin protein is associated with internal membrane disruption, while o-spanin is associated with the disruption of the external membrane [41]. Importantly, spanins are phage lysis proteins that act together to form a bridge between i-spanin and o-spanin [42]. While both o-spanin and i-spanin were found in vB_Si_35FD, in phage vB_Si_DR094, only the i-spanin gene was found. Further studies are necessary to understand the function of spanins in these phages.

Moreover, two rII lysis inhibitors were found in both phages. Studies of phage T4 describe the function of these inhibitors in the integration of the loci r (rapid lysis) [43]. Padisson (1998) studied these genes and concluded that only rI, rIII, and rVI are directly related to the inhibition of lysis, which is a process that leads to the accumulation of viral particles inside the bacterial cell, increasing its size [44]. Therefore, these genes do not cause the direct inhibition of the lysis process, suggesting that the phage-driven lysis of bacteria is achieved through the participation of different genes. In consequence, while rII lysis inhibitors have been previously described for *Felixounaviruses*, their function has not yet been elucidated [28]. An additional genomic region of approximately 15 kb was observed that includes only hypothetical proteins (Figure 1C,D).

We found that both genomes did not present genes associated with bacterial virulence or antimicrobial resistance that could be transferred to another hosts, or genes indicative of a possible integration into the bacterial genome (e.g., integrases or transposons). These results support the lytic reproduction cycle, and the lack of the integration machinery are important characteristic as possible biocontrol agents. In addition, a phylogenetic analysis was conducted to evaluate the closeness between the large subunit terminase of FelixOVT1 and the two new phages reported here (Supplementary Figure S1). The inferred phylogeny showed that phages vB_Si_35FD and vB_Si_DR94 are related to FelixOVT1 in terms of the genome packaging mechanism [28]. However, other studies, e.g., of the stability of these phages in different conditions and of the multiply of infection in an innocuous host, are necessary [45,46].

The complete taxonomic classification of phages vB_Si_35FD and vB_Si_DR094 was carried out using an alignment utilizing BLAST with phage FelixO1 (accession number AF320576.1), which is the type species for the genus *Felixounavirus*. We observed an aminoacidic identity for vB_Si_35FD and vB_Si_DR94 of 97.70% and 96.64%, respectively, and a nucleotide identity of 97.32% and 97.00%, respectively; thus, these two phages belong to the *Felixounavirus* genus [47].

### 2.2. Comparative Analysis of Felixounavirus that Infect Salmonella

#### 2.2.1. *Felixounavirus* that Infect *Salmonella* are Highly Similar on a Global Scale

Genomes were selected from the NCBI database (https://www.ncbi.nlm.nih.gov/, accessed on 1 June 2020) using a filter based on the term *Felixounavirus* that resulted in 73 genomes. Subsequently, a second filter was applied with the term *Salmonella*, generating 23 whole genomes, in addition to both phages described in this work (Table 1). In general terms, these genomes represented phages that were isolated from different sources, mostly (9/25) from animal feces, while others were recovered from sewage samples at different facilities (e.g., layer house). Phage Mushroom (KP143762.1) was the only member contained in a commercial kit (IntestiPhage), which was developed by the Georgia Eliava Institute of Bacteriophages, Microbiology and Virology, Tbilisi, Georgia [26,48].

Regarding the geographical origin of these phages, they were isolated from three different continents: the Americas (the US and Chile), Asia (Russia and China), and Europe (Georgia, Spain, and Switzerland), with China reporting 7/23 of the phage genomes from the genus *Felixounavirus*. This genus is distributed worldwide, with different sources used for isolation, and animal feces as one of the most frequent sources. The fact that animals are carriers of *Salmonella* with high levels of antimicrobial resistance has encouraged studies of screening for phages, as alternative strategies for controlling this pathogen [49]. Moreover, *Felixounavirus* related to *Salmonella* were found with desirable characteristics and selected for sequencing. The genome sizes of all phages reported is on average 86.6 kb, with phage ST11 (MF370225.1) containing the smallest assembled genome of 82.1 kb and phage BPS15Q2 the largest assembled genome of 89.8 kb (KX405003.1) (Table 1). Re-annotation of the 23 analyzed genomes revealed that the amounts of tRNA ranged from 17 to 23 and coding regions (CDS) ranged from 125 to 165, among which phage GE_vB_7A isolated from Georgia contained 165 CDS, yet still did not represent the largest assembled genome. 

**Figure 2:**
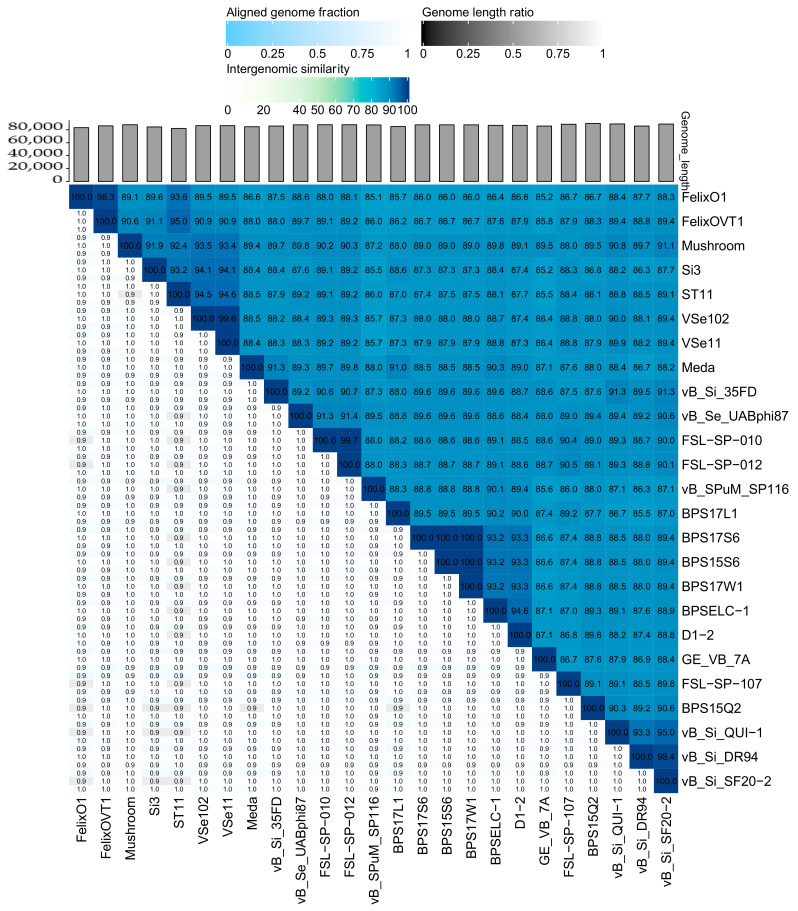
Heat map generated by VIRIDIC [47]. The right side shows the intergenomic similarity between the 25 genomes using a gradient in blue, with darker colors showing a greater percent of intergenomic similarity. The left half represents three different values, the aligned genome fraction for the genome found in the row (on the top), genome length ratio (in the middle), and the aligned genome fraction (on the bottom) for the genome found on the column, all ranked from 0 to 1, with 1 represented in white. The genome length for all phages was 80 kp.

Nucleotide-based intergenomic analysis showed similarities above 85.1%, with an aligned fraction of the genome of 0.9-1.0 and a genome length ratio of 0.9–1.0 (Figure 2). The thresholds used to group phages in taxonomic levels are 70% for genus and 95% for species [47]. The two new members reported here were 89.5% similar (Figure 2). A total of 10 phages are represented in the same species, of which the phages vB_Si_DR94 and vB_Si_SF20-2 showed 98.4% similarity. This was also noted with phages vB_Si_QUI-1 and vB_Si_SF20-2, which have a similarity of 95.0% and were also recovered in Chile from poultry feces [19]. On the other hand, phages VSe102 and ST11 had 99.6% similarity and both viruses were isolated in Russia [17]. Interestingly, the phage ST11 and FelixO1_VT1 showed 95.0% similarity but were isolated from different countries. Specifically, phage ST11 was isolated in Russia from chicken feces, whereas phage FelixO1_VT1 was collected in the US with source unreported [50]. Likewise, we observed a cluster of three phages with 100% similarity (BPS17S6, BPS15S6, and BPS17W1) (Figure 2). All of these phages were isolated in China from sewage samples in layer houses or slaughterhouses (Table 1). Overall, our findings show that the genus *Felixounavirus* is composed of highly similar members with a wide geographical distribution.

**Figure 3:**
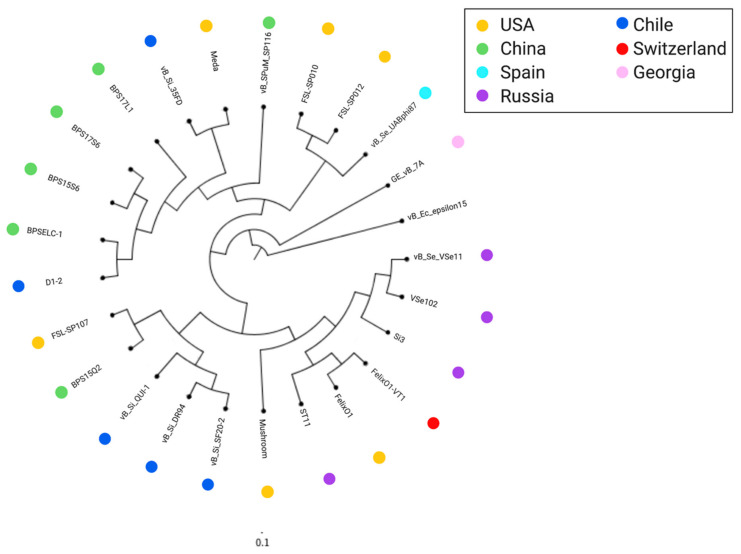
Phylogenetic tree of phage vB_Si_35FD, vB_Si_DR94 and the 23 genomes downloaded from NCBI (rooted with vB_Ec_epsilon15). Three clades were observed: Clade A (light grey) with 7 phages, mostly from Russia (purple circles). Clade B (medium grey) contains 5 phages, mostly isolated from Chile (blue circles). Clade C (dark grey) has 11 phages, with a subclade of phages isolated from China (green circles). The singleton phage vB_St_GE7A was isolated from Georgia (pink circle). Phage vB_Ec_epsilon15 is the outgroup, it was not compared with the other *Felixounavirus* genomes compared in Figure 2.

Phylogenetic analysis was carried out with VICTOR to evaluate the relationships among the *Salmonella*-infecting *Felixounavirus*. We found three main clades (Figure 3): (i) clade A, which contains the type species FelixO1 along with other phages isolated mostly in Russia, (ii) clade B that contains five phages, including the new member vB_Si_DR094, one phage isolated in China, one in the US, and another three phages isolated in Chile from animal feces using *Salmonella* Infantis as a host [19] (Table 1). Moreover, (iii) clade C contains the phage vB_Si_35FD and 10 other phages. Interestingly, one subclade here contains only phages isolated in China (BPSLEC-1, BPS15S6, BPS17S6, and BPS17L1). Phage vB_Si_35FD was grouped with phage Meda, isolated in the US from a soil sample in a cattle holding pen using *Salmonella* Heidelberg as a host [18]. Within the tree, phage GE_vB_7A stands out as a singleton in a cluster phylogenetically distant from the rest of the genomes. This phage was isolated using *Salmonella* Typhimurium as a host from Mtkvari river water in Georgia [51]. Overall, this analysis showed two scenarios, one in which closely related *Felixounavirus* that infect *Salmonella* were obtained from very distant geographical locations and the other in which closely related phages were obtained in close geographical locations. Their host *Salmonella* spp. is a worldwide pathogen, thus, it is expected that these phages would also have a worldwide distribution, although it is important to note their conserved genomes. As *Salmonella* is transmitted through food around the globe, it is tempting to think that *Felixounavirus* that infect *Salmonella* are also disseminated worldwide by the food trade. Additionally, there is a study that tested *Salmonella* isolated from different countries against three different phage cocktails from the Eliava Institute. Three of these phages belong to the *Felixounavirus* genus, and all of the strains were susceptible to at least one phage. These results suggest that their worldwide distribution and different sources might not necessarily interfere with the effectiveness of these phages [20].

#### 2.2.2. Similar Genomic Backbone in *Felixounaviruses* that Infect *Salmonella*

**Figure 4:**
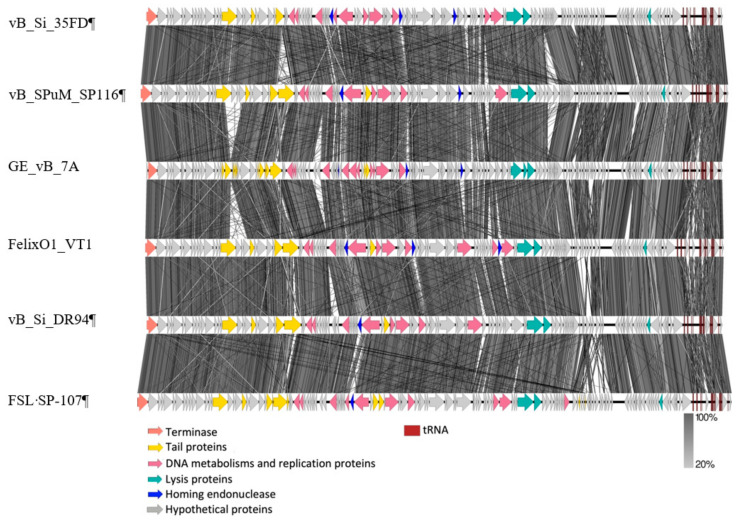
Aminoacidic alignment of six phages. The genes with relevance were marked with colors. All genomes start from terminase large subunits (orange), tail proteins were marked in yellow, all phages presented common components, such as tail tape measure proteins. Proteins associated with DNA metabolism and replication were marked in pink and lysis proteins in green. All phages contained two proteins associated lysis. Homing endonucleases were marked in blue and hypothetical proteins in grey. tRNAs were found at the end of all genomes (dark red squares).

Six genomes, one from clade A (FelixO1), two from clade B (vB_Si_DR94 and FSL-SP107), two from clade C (vB_SPuM_SP116 and vB_Si_35FD), and the singleton (GE_vB_7A) were further aligned and displayed using EasyFig (Figure 4). Overall, a very similar transcriptional modular order was observed in all genomes, despite the phylogenetic distance described above. In general, the proteins were grouped by function, such as those involved in structure or metabolism (Figure 4), as described above for the two new members of the genus. A great proportion of the genome was formed by genes encoding proteins involved in DNA metabolism (indicated in pink) (Figure 4). The six genomes had in common enzymes involved in DNA metabolism, such as thymidylate synthase, dihydrofolate reductase, exodeoxyribonuclease, deoxyribonuclease monophosphate kinase, and proteins associated with replication such as DNA polymerase, DNA ligase, and DNA primase/helicase. Phage FSL-SP107 had a putative integration and excision endonuclease VII (ACLAME 151), which has been described in the phage T4 as a key component of the mismatch repair mechanism [52].

Other relevant characteristics include the fact that the six genomes displayed some diversity in their tail tape measure proteins that affect the length of the tail. Some differences were detected in the genes associated with the tail fibers, which are also known as RBPs (receptor binding proteins) that recognize specific receptors on the bacterial surface, which drives the phage’s host range [53]. As mentioned above, *Felixounavirus* phages contain wide host ranges affecting multiple *Salmonella* serovars. As previously mentioned, the bacterial receptor for this genus corresponds to LPS, a molecule with a high rate of variability [54,55]. Importantly, the tail fibers presented considerable diversity among the phage genomes analyzed in this work (Figure 4). Since tail fibers undergo constant changes, the bacterial hosts show several modifications of their receptors in order to generate immunity against phages, and, in response, phages tend to present mutations that enable recognizing the new modified receptor, which represents a co-evolutionary process [56]. Consequently, in the same genus, variations in the tail fibers are expected, which arise upon exposure to their host [19].

In conclusion, *Felixounaviruses* that infect *Salmonella* obtained from distinct geographical areas are not only similar at the nucleotide level, but also in their overall genome synteny. To better understand the diversity and stability of these phages, collections from around the globe need to be analyzed with identical *Salmonella* host strains in addition to analyses of the differences found in the genomes regarding host range and other phenotypes. All of these data will help to achieve a better understanding of the main characteristics of these phages, which at this point, in a genomic vision, appear to have potential to be used as strategy to control *Salmonella* in the food production chain. While the genomes analyzed in the present study represent a very small number and do not represent their overall global diversity, our results support a genome stability of *Felixounavirus* phage infecting *Salmonella* that warrants further testing, with more availability of *Felixounaviruses* that infect *Salmonella* genomes.

## 3. Materials and Methods

### 3.1. Bacteria and Phage Growth Conditions 

Four *Salmonella* isolates from different serovars were used to isolate bacteriophages: *S.* Enteritidis (FSL S5-371), *S.* Infantis (FSL S5-506), *S.* Heidelberg (FSL S5-455), and *S.* Typhimurium (FSL S5-370). All isolates were obtained from the Food Safety Laboratory (FSL; Cornell University, Ithaca, NY, USA). *Salmonella* isolates were incubated in tryptic soy broth (TSB, BD Difco, Franklin Lakes, NJ, USA) at 37 °C for 16 h. Two phages were characterized in this study: phage *vB_Si_35FD*, for which the isolation was previously reported [57], and phage vB_Si_DR094 that was recovered from a cow fecal sample in Easter Island, using a previously described protocol [19]. Both phages were propagated in *S*. Infantis (FSL S5-506). A total of 300 uL from an overnight culture of FSL S5-506 and 100 uL from the phage stock were mixed in 4 mL of 0.75% tryptic soy agar (TSA) and poured in TSA plates [19]. Phage stocks were tittered using previously described protocols [19] and lysates were maintained at 4 °C.

### 3.2. Host Range Characterization Phages vB_Si_35FD and vB_Si_DR94

For both phages we studied the host range as described previously by Rivera et al., 2018 [19], including the same 26 *Salmonella* hosts. Shortly, we spotted 5 uL of phage lysate over a host cell lawn prepared with 1:10 dilution of the overnight culture of the host strain in 4 mL of 0.75% tryptic soy agar (TSA) and poured in TSA plates. The plates were incubated for 16 h at 37 °C and then examined for lysis.

### 3.3. Morphological Characterization

Phage vB_Si_35FD was further subjected to morphological characterization by transmission electron microscopy (TEM). Phage lysate of vB_Si_35FD was precipitated with polyethylene glycol PEG8000 (Sigma-Aldrich, Saint Louis, MO, US). Solutions were prepared for each phage at a concentration of 10^11^ PFU/mL. The sample was deposited on 300-mesh carbon-coated FORMVAR copper grids, then negatively stained with 2% uranyl acetate for 40 s [57,58,59]. The samples were observed at 80 kV with TEM Phillips Tecnai 12 (Biotwin; Quebec, Canada) in the Advanced Microscopy Unit of the Catholic University of Chile. TEM of phage vB_Si_DR094 was prepared by floating a glow discharged 40- mesh copper grid coated with a thin carbon film on cesium chloride-purified phage samples at 10^11^ PFU/mL then stained with 2% uranyl acetate. Samples were observed at 120 kV using a Tecnai G2 Spirit BioTWIN TEM, and the images were captured using an Eagle TM 2k CCD. Electron microscopy was performed at the Characterization Facility of the University of Minnesota.

### 3.4. DNA Extraction and Sequencing

Phenol/chloroform DNA extraction was performed for phage vB_Si_35FD according to [37] and precipitated with ethanol. The DNA concentration was determined by OD measurement with a Maestro Nano Pro-Spectrophotometer (Maestrogen Inc., Hinschu, Taiwan) and the quality was determined according to the 260/280 nm ratio. Sequencing libraries and sequencing were conducted at MicrobesNG (Birmingham, United Kingdom). 

vB_Si_DR094 DNA was sequenced by Laragen, Inc. (Los Angeles, CA, US) using Illumina MiSeq whole-genome sequencing followed by Contig assembly. DNA was isolated using the Phage DNA Isolation Kit (Product #46800), Norgen BioTek Corporation (Ontario, Canada) from cesium chloride-purified vB_Si_DR094 phage.

### 3.5. Genome Annotation

Annotation was performed using RASTtk for both phages [60]. Putative protein-encoding open reading frames were identified using Prodigal and Glimmer as an argument within the RASTtk pipeline. With the annotated bacteriophage sequences, the functional assignments were manually conducted with Blastp (https://blast.ncbi.nlm.nih.gov/Blast.cgi, accessed on 1 August 2020). The tRNAs were annotated using tRNAscan-SE v2.0 included in RASTtk [61]. Phage genome maps were prepared with the GCView server using the default settings [62]. 

### 3.6. Genome Sequence Accession Number

The annotated genomic sequences for phages vB_Si_35FD and vB_Si_DR94 are available from the NCBI database under the accession numbers MZ327261 and MZ327262, respectively.

### 3.7. Selection of Genomes and Comparative Analysis

Genomes were selected from the NCBI database and filtered first using the term *Felixounavirus*, followed by a second filter of *Salmonella* spp. resulting in 23 genomes (Table 1). Subsequently, a dataset was created with the selected genomes, and each of the genomes contained in this dataset was annotated with the bioinformatic tool RASTtk [60]. With the annotation of all genomes, large terminase subunit genes were mapped and re-oriented using this gen as a start, and then the genomes were annotated again with RASTtk. 

Phylogenetic analyses were conducted using VICTOR (https://ggdc.dsmz.de/victor.php, accessed on 1 October 2020), using the default settings [63]. All pairwise comparisons of the amino acid sequences were conducted using the Genome-BLAST Distance Phylogeny (GBDP) method [64] with the settings recommended for prokaryotic viruses [63]. The resulting intergenomic distances were used to infer a balanced minimum evolution tree with branch support via FASTME including SPR postprocessing [65] for each of the formulas D0, D4, and D6. Branch support was inferred from 100 pseudo-bootstrap replicates each. The trees were rooted at the midpoint [66] and visualized with FigTree [67]. Taxon boundaries at the species, genus, and family levels were estimated with the OPTSIL program [68], the recommended clustering thresholds [53], and an F value (fraction of links required for cluster fusion) of 0.5 [69]. The phage vB_Ec_e15 infecting *Escherichia coli* was used as a tree root.

Four genomes were selected from the phylogenetic tree: vB_SPuM_SP116 (KP010413.1), GE_vB_7A (MG969404.1), FSL-SP107 (KC139638.1 and KC139640.1), and FelixO1 (AF320576.1) for the phylogenetic analyses, along with the phages vB_Si_35FD and vB_Si_DR094. These were aligned using EasyFig v2.2.2 [70]. The average BLAST nucleotide identities were calculated by using VIRIDIC [47].

Phylogenetic analyses for the large subunit of the terminase were performed by using the Neighbor-Joining method [71]. The percentage of replicate trees was shown in which the associated taxa clustered together in the bootstrap test (1000 replicates) [72]. The evolutionary distances were computed using the p-distance method [73] and are in units of the number of amino acid differences per site. The rate variation among sites was modeled with a gamma distribution (shape parameter = 1). This analysis involved 4 amino acid sequences. All positions with less than 95% site coverage were eliminated, i.e., fewer than 5% alignment gaps, missing data, and ambiguous bases were allowed at any position (partial deletion option). There was a total of 391 positions in the final dataset. Analyses were conducted in MEGA X [74].

## 4. Conclusions

This study describes two new members of the *Felixounavirus* genus that infect *Salmonella* Infantis. Genomic comparison of 25 genomes suggests that, despite being isolated from different sources and geographic regions, the phages share a high level of genome identity and synteny. These results contribute to the understanding of *Felixounavirus* phages that infect *Salmonella*, which is of importance given that these phages have the potential to serve as a means of biocontrol for *Salmonella*.

## Figures and Tables

**Table 1:** Genome information for all genomes selected for comparative analysis.

Phage	Accession Number	Genome Size (kb) *	%GC *	CDS *	tRNA *	Isolation Country	Isolation Sample
FelixO1	JF461087.1	83.33	38.9	125	18	Switzerland	-
Mushroom	KP143762.1	87.71	39	129	22	USA	IntestiPhage
D1-2	MN481367.1	86.88	38.7	132	18	China	Sewage
vB_Si_SF20-2 (DaR-2019b)	MK965970.1	88.97	39.1	131	20	Chile	Poultry feces
vB_Si_QUI-1 (DaR-2019a)	MK965969.1	89.09	39.1	129	20	Chile	Poultry feces
Meda	MH586731.1	84.67	38.8	131	19	USA	Soil in the cattle holding pen of cattle harvest facility
GE_vB_7A	MG969404.1	85.78	39.0	165	21	Georgia	Mtkvari river water
BPS17W1	MG646669.1	87.61	38.8	130	19	China	Sewage samples from hog house
BPS17S6	MG646671.1	87.63	38.8	131	19	China	Sewage samples from layer house
BPS17L1	MG646672.1	84.92	38.9	125	21	China	Sewage samples from slaughterhouse
BPS15S6	MG646670.1	87.61	38.8	130	19	China	Sewage samples from layer house
VSe102	MG251392.1	86.37	39.0	126	17	Russia	Farm sewage
VSe11	MG251391.1	86.36	39.0	126	17	Russia	Sewage
ST11	MF370225.1	82.1	39.0	130	19	Russia	Chicken feces
Si3	KY626162.1	84.42	39.0	125	17	Russia	-
BPS15Q2	KX405003.1	89.82	38.9	132	20	China	Domestic sewage samples
vB_SPuM_SP116	KP010413.1	87.51	38.8	130	21	China	Sewage
FelixO1VT	AF320576.1	86.16	39.0	126	19	USA	-
BPSELC-1	MN227145.1	86.99	38.8	129	19	China	Chicken manure
FSL-SP-010	** KC139526.1-KC139527.1-KC139528.1	87.73	39.1	134	18	USA	Bovine feces
FSL-SP-012	** KC139543.1-KC139544.1	87.81	39.0	132	19	USA	Bovine feces
FSL-SP-107	** KC139640.1-KC139638.1	88.52	39.0	136	19	USA	Bovine feces
vB_Si_35FD	MZ327261	85.81	38.9	129	19	Chile	Bovine feces
vB_Si_DR094	MZ327262	85.7	39.2	125	20	Chile	Bovine feces
vB_Se_ UABphi87	NC_027360.1	87.8	38.9	129	23	Spain	-

* The information on genome sizes, %GC, CDS, and tRNAs was obtained from RASTtk annotation, and the accession numbers, isolation countries, and samples were obtained from the NCBI database. ** For these phages, pseudo genomes uploaded to the NCBI database with those accession numbers were used, assembled, and annotated with RASTtk.

## Data Availability

Sequences generated in this study are publicly available at ncbi.

## Supplementary Materials

The following are available online at https://www.mdpi.com/article/10.3390/antibiotics10070806/s1, Figure S1: Phylogenetic tree of the large subunit of terminase.
