# Supplementary material for: Comparative Analysis of Felixounavirus Genomes Including Two New Members of the Genus That Infect Salmonella Infantis
Source: Antibiotics (Basel). 2021 Jul 2;10(7):806. doi: 10.3390/antibiotics10070806 (PMC8300805; doi:10.3390/antibiotics10070806)
Supplement: Supplementary file 1 [file antibiotics-10-00806-s001.zip › antibiotics-1277196-supplementary.pdf]

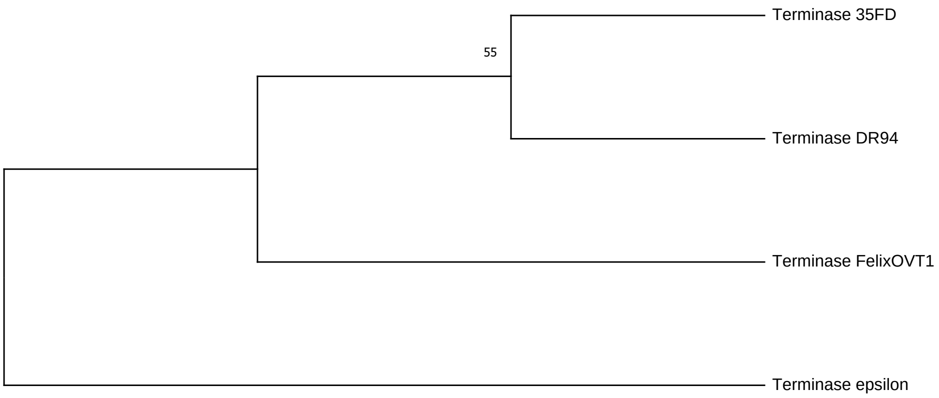

Supplemental Figure 1: Phylogenetic tree of the large subunit of terminase for phages vB_Si_35FD, vB_Si_DR94 and FelixOVT1. Phage vB_Ec_epsilon15 is the outgroup. Bootstrap test (1000 replicates).
